# Beyond Kyphosis: A Case of Tuberculous Spondylodiscitis With L3 Vertebral Dislocation

**DOI:** 10.7759/cureus.113593

**Published:** 2026-07-29

**Authors:** Nitin Pothare, Sushil Mankar, Ronit P Dalvi, Aditya Patel, Swapnil Thorat

**Affiliations:** 1 Orthopedic Surgery and Spine Surgery, Medinine Hospital, Nagpur, IND; 2 Spine Surgery, N.K.P. Salve Institute of Medical Sciences & Research Centre and Lata Mangeshkar Hospital, Nagpur, IND; 3 Orthopedics and Traumatology, N.K.P. Salve Institute of Medical Sciences & Research Centre and Lata Mangeshkar Hospital, Nagpur, IND; 4 Orthopedic Surgery, N.K.P. Salve Institute of Medical Sciences & Research Centre and Lata Mangeshkar Hospital, Nagpur, IND; 5 Orthopedics, N.K.P. Salve Institute of Medical Sciences & Research Centre and Lata Mangeshkar Hospital, Nagpur, IND

**Keywords:** lateral lumbar corpectomy, pott's disease, pott's paraplegia, tuberculous spondylitis, vertebral collapse, vertebral lesion

## Abstract

Spinal tuberculosis (TB) accounts for the majority of skeletal TB cases and remains a significant cause of morbidity in developing nations. While thoracic involvement is most common, lumbar vertebral dislocation represents an exceedingly rare and severe complication. This case involved a 22-year-old female who presented with a six-month history of severe back pain followed by the sudden onset of complete paraplegia with bowel and bladder incontinence. Despite initiation of antitubercular therapy (ATT) at another facility, her condition progressively worsened. Neurological examination revealed flaccid paralysis of both lower limbs and complete sensory loss below the L3 level. Imaging confirmed L3 vertebral body destruction with anterior dislocation consistent with tuberculous spondylitis and spinal instability. Surgical intervention was performed via an anterior retroperitoneal approach, including L3 corpectomy, debridement of infected tissue, and anterior column reconstruction using an autologous iliac crest bone graft. This case illustrates a rare presentation of tuberculous spondylodiscitis with vertebral dislocation and highlights the importance of early diagnosis and timely surgical intervention in patients with progressive neurological deficits and spinal instability. While ATT remains the cornerstone of treatment, surgical decompression and stabilization are imperative in cases of significant mechanical instability to prevent further neurological deterioration.

## Introduction

Tuberculosis (TB) of the spine remains a notable cause of morbidity, particularly in developing nations [1]. Approximately 50% of skeletal TB cases involve the spine [2]. Although the thoracic spine is more commonly affected, lumbar involvement with vertebral dislocation is exceedingly rare [3]. This report describes a case of L3 vertebral dislocation due to spinal TB that resulted in paraplegia and was managed surgically via an anterior approach.

## Case presentation

**Figure 1:**
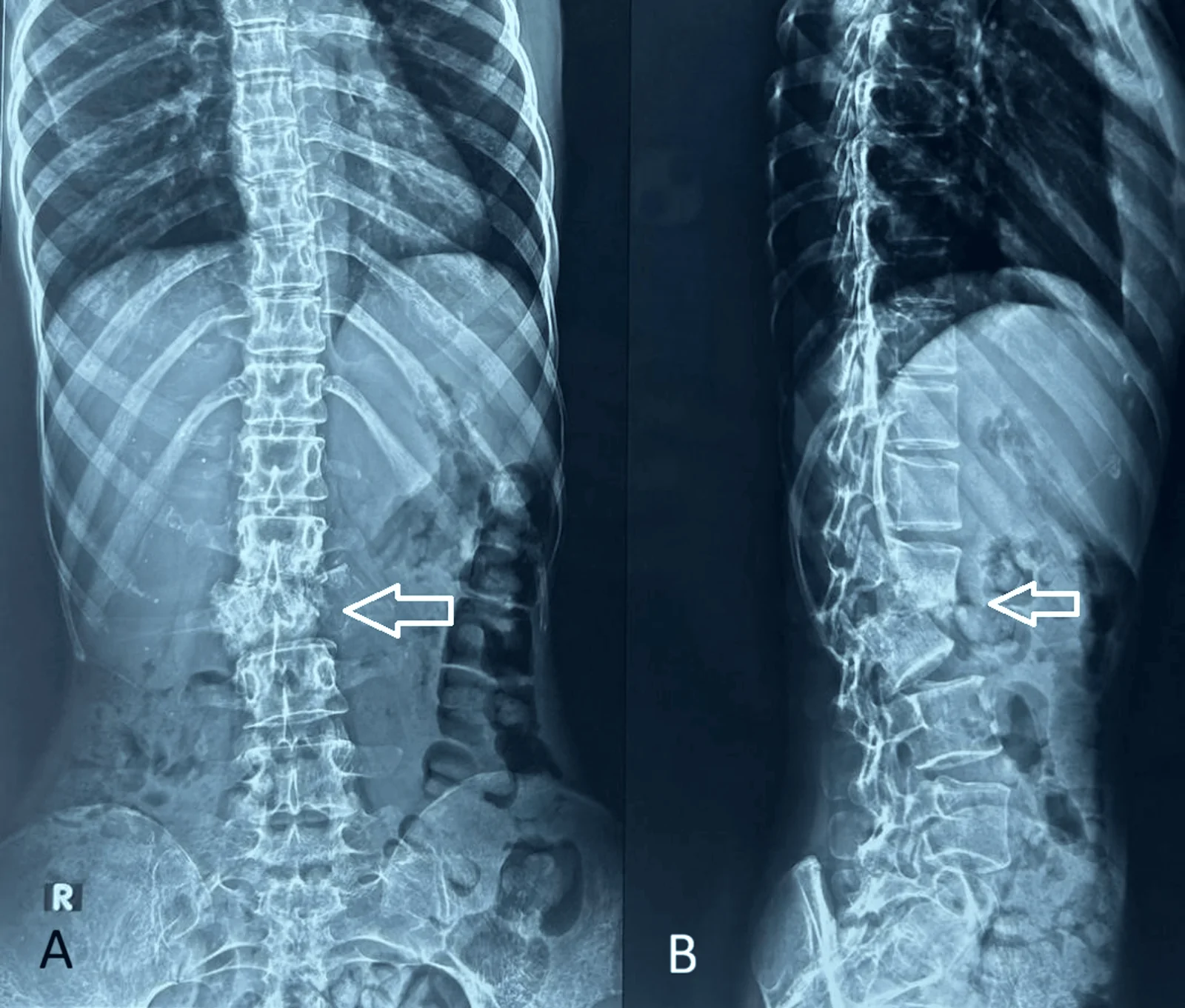
Radiographs of the thoracolumbar spine (A, anteroposterior; B, lateral views) showing destruction and collapse of the L2 and L3 vertebrae.

A 22-year-old female presented with a six-month history of severe back pain, followed by the sudden onset of bilateral lower limb weakness. She had been previously diagnosed with spinal TB and started on antitubercular therapy (ATT). Despite the initiation of treatment, her condition progressively worsened, leading to complete paralysis of the lower limbs and bowel and bladder dysfunction. On neurological examination, both lower limbs demonstrated complete flaccid paralysis. Clinically, bilateral hip, knee, and ankle muscle power was 0/5. Deep tendon reflexes were absent, and plantar reflexes were mute bilaterally. Sensory examination revealed complete loss of sensation below the L3 level. The patient also had bowel and bladder incontinence. Radiological examination was performed, and digital radiographs of the thoracolumbar spine revealed destruction and collapse of the L2 and L3 vertebrae (Figure 1).

**Figure 2:**
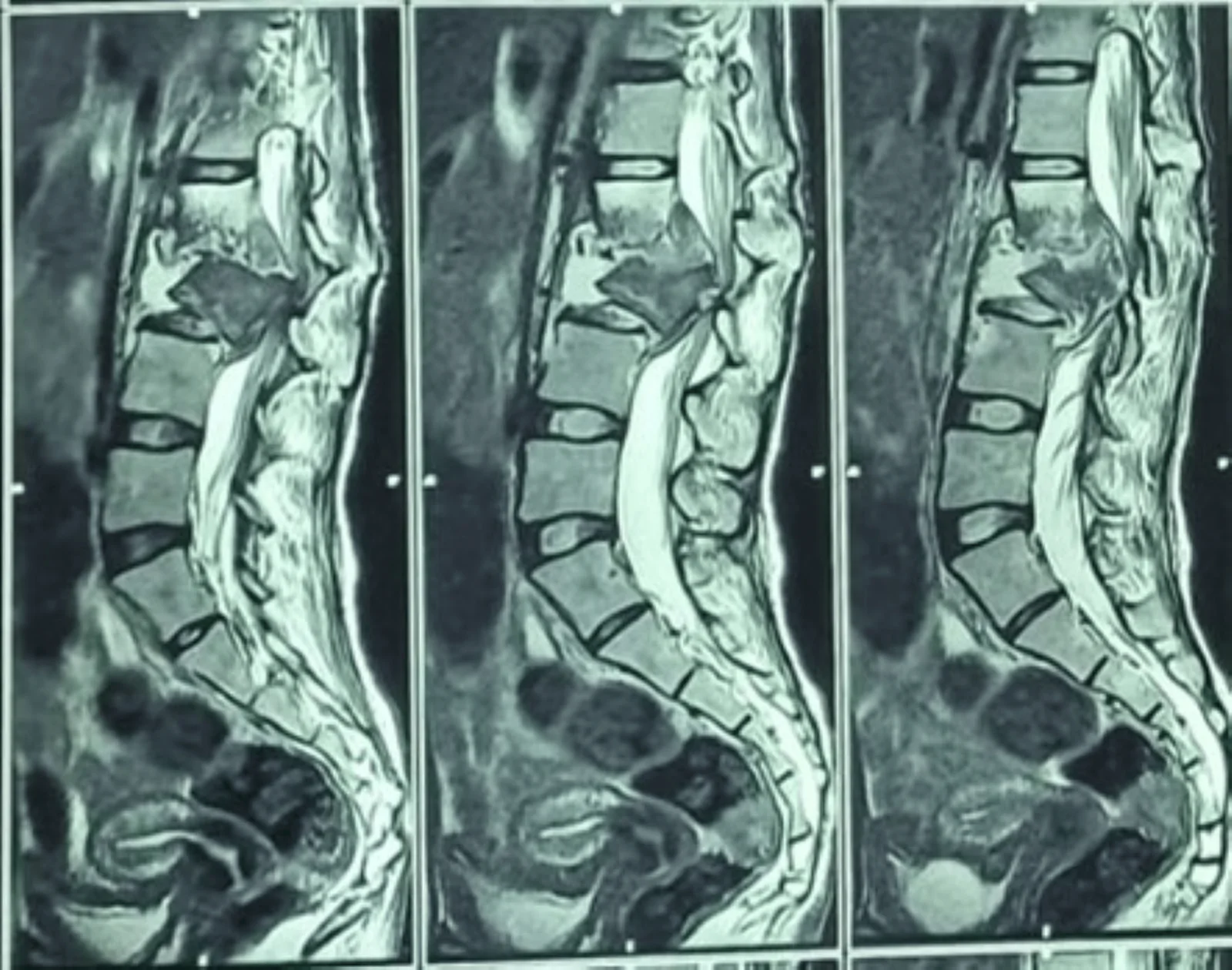
MRI of the thoracolumbar spine showing L3 vertebral collapse with retropulsion and posterior element involvement in a background of diffuse T2 hypointense marrow, favoring chronic healed tuberculous spondylitis with resultant instability and reduced spinal canal space.

An MRI was performed and showed L3 vertebral collapse with retropulsion and posterior element involvement in a background of diffuse T2 hypointense marrow, consistent with chronic healed tuberculous spondylitis with resultant instability and reduced spinal canal space (Figure 2).

Surgical procedure

An anterior approach was selected because of its feasibility and the better control it provides for decompression and reconstruction. The patient was positioned in the left lateral decubitus position, and the operating table was adjusted to increase exposure through the iliac crest and the costal margin. An incision was made along the 12th rib, and a retroperitoneal approach was used. The lumbar fascia and abdominal musculature were carefully retracted, followed by gentle mobilization of the psoas muscle, with identification and preservation of the ureter. An L3 corpectomy was then performed to remove the diseased vertebral body, and an autologous iliac crest bone graft was harvested and inserted to achieve anterior column reconstruction.

Postoperative protocol

In the immediate postoperative period following spinal TB fixation surgery, the patient’s vital signs, neurological status, wound condition, and drain output were closely monitored. Adequate pain management was achieved using appropriate analgesia, with a gradual transition from intravenous to oral medications. Perioperative antibiotics were continued according to institutional protocol. ATT was continued based on the clinical and radiological response. Nutritional rehabilitation with a high-protein, high-calorie diet, along with vitamin and mineral supplementation, was initiated to promote recovery and wound healing. Early mobilization was encouraged once the patient became hemodynamically stable, beginning with log-rolling techniques and bedside sitting with support. A thoracolumbosacral brace was used to provide additional spinal stabilization during the early recovery period. Physiotherapy included respiratory exercises, limb range-of-motion exercises, and deep vein thrombosis prevention measures. As neurological recovery progressed, focused neurorehabilitation was continued to improve independence. Regular clinical, neurological, laboratory, and radiological follow-up was performed to assess implant integrity, spinal fusion, deformity correction, and response to ATT. In view of the patient’s neurological deficit, comprehensive spinal cord rehabilitation, including bladder and bowel care, pressure sore prevention, mobility training, and psychological support, was also incorporated to optimize long-term functional recovery and quality of life.

## Discussion

Tuberculous spondylodiscitis remains one of the most common forms of extrapulmonary TB and continues to be a significant cause of spinal deformity and neurological compromise, particularly in developing countries [4,5]. It typically involves the anterior column of the spine, leading to progressive vertebral body destruction, disc space narrowing, and kyphotic deformity. However, gross vertebral dislocation, as observed in our case at the L3 level, is an exceptionally rare and severe manifestation of the disease.

The pathophysiology of spinal TB involves hematogenous dissemination of *Mycobacterium tuberculosis *to the vertebral bodies, most commonly affecting the paradiscal region [2,6]. Progressive caseous necrosis and osteolysis result in weakening of the vertebral structure, ultimately leading to collapse. In advanced cases, destruction of the anterior and middle columns may lead not only to angular kyphosis but also to translational instability and vertebral dislocation. Paraplegia in our patient may have been due to compression of the spinal cord or cauda equina resulting from the significant instability.

In spinal TB, neurological deficits may result from several mechanisms. Important causes include mechanical compression by an abscess, granulation tissue, or bony fragments, as well as vascular compromise and inflammatory edema. In this case, the profound neurological impairment was due to severe vertebral destruction with anterior dislocation, which led to both mechanical and ischemic neural injury.

MRI, the gold standard imaging modality for the diagnosis of spinal TB, was used and allowed meticulous evaluation of vertebral involvement, extension into the paraspinal soft tissues, and compression of neural structures [7]. Typical MRI findings include hypointense vertebral bodies on T1-weighted images, hyperintensity on T2-weighted images, disc involvement, and the presence of paravertebral or epidural abscesses. However, in advanced cases such as this, MRI may reveal vertebral collapse with displacement, making it more difficult to characterize instability patterns and plan the surgical intervention.

Management of tuberculous spondylodiscitis primarily involves prolonged ATT, which remains the cornerstone of treatment [8]. However, surgical management is advised in cases of progressive neurological deficit, spinal instability, severe deformity, and failure of medical management. The presence of vertebral dislocation, as in our patient, represents a clear indication for surgical stabilization because of the inherent mechanical instability and high risk of further neurological deterioration.

The surgical approach in such cases aims to achieve adequate decompression of the neural elements, debridement of infected tissue, correction of deformity, and stabilization of the spinal column. Various approaches, including anterior, posterior, or combined techniques, have been described depending on the extent and location of the disease. In our case, surgical management was essential not only to address the neurological deficit but also to restore spinal alignment and stability.

This case highlights the diagnostic and therapeutic challenges associated with advanced spinal TB. The presence of vertebral dislocation is a rare but critical complication that underscores the aggressive nature of the disease when diagnosis and treatment are delayed. Early recognition and timely initiation of ATT are crucial in preventing such severe sequelae.

From a clinical perspective, this case emphasizes the need to consider spinal TB in the differential diagnosis of patients presenting with back pain and neurological symptoms, especially in endemic regions. Furthermore, it illustrates that atypical and severe presentations, including vertebral dislocation, can occur and require prompt surgical intervention.

## Conclusions

This case highlights the importance of early identification of aggressive spinal TB, especially in patients who present with severe or progressive neurological deficits and atypical radiological findings. Careful assessment of the clinical findings in conjunction with imaging is essential for accurately assessing the extent of disease and facilitating timely management decisions.

Although ATT forms the cornerstone of treatment, surgical intervention becomes imperative in the presence of significant neurological compromise and mechanical instability. Prompt decompression and stabilization can be crucial in preventing further neurological deterioration and improving functional outcomes. Increased awareness of such rare presentations is essential for spine surgeons to enhance diagnostic accuracy and optimize patient care.
